# Elevated arousal at time of decision-making is not the arbiter of risk avoidance in chickens

**DOI:** 10.1038/srep08200

**Published:** 2015-02-03

**Authors:** A. C. Davies, A. N. Radford, I. C. Pettersson, F. P. Yang, C. J. Nicol

**Affiliations:** 1 Animal Welfare and Behaviour Group, School of Clinical Veterinary Science, University of Bristol, Langford House, Langford, Bristol, BS40 5DU, UK; 2 School of Biological Sciences, University of Bristol, Life Sciences Building, 24 Tyndall Avenue, Bristol, BS8 1TQ, UK; 3 College of Bioscience and Biotechnology, Yangzhou University, Yangzhou, Jiangsu, China, 225009

## Abstract

The somatic marker hypothesis proposes that humans recall previously experienced physiological responses to aid decision-making under uncertainty. However, little is known about the mechanisms used by non-human animals to integrate risk perception with predicted gains and losses. We monitored the behaviour and physiology of chickens when the choice between a high-gain (large food quantity), high-risk (1 in 4 probability of receiving an air-puff) option (HGRAP) or a low-gain (small food quantity), no-risk (of an air-puff) (LGNAP) option. We assessed when arousal increased by considering different stages of the decision-making process (baseline, viewing, anticipation, reward periods) and investigated whether autonomic responses influenced choice outcome both immediately and in the subsequent trial. Chickens were faster to choose and their heart-rate significantly increased between the viewing and anticipation (post-decision, pre-outcome) periods when selecting the HGRAP option. This suggests that they responded physiologically to the impending risk. Additionally, arousal was greater following a HGRAP choice that resulted in an air-puff, but this did not deter chickens from subsequently choosing HGRAP. In contrast to human studies, we did not find evidence that somatic markers were activated during the viewing period, suggesting that arousal is not a good measure of avoidance in non-human animals.

An important aspect of adaptive decision-making is the ability to use prior experiences to assess potential gains and losses, thus making predictions about optimal choices^1^. Under natural conditions, trade-offs between high-gain, high-risk options and low-gain, low-risk options often exist and individuals have been shown to alter their behaviour in response to a wide variety of risks, including predation^2^, variation in food quality^3^, environmental temperature^4^ and flight collisions^5^. However, despite the importance of risk assessment in adaptive decision-making, we know relatively little about the mechanisms used by non-human animals to integrate their perceptions of risk with likely gains and losses.

Decisions must frequently be made rapidly with imperfect knowledge of the available options, making it impossible for an individual to “weigh-up” the costs and benefits of decision outcomes accurately^6^. Under such conditions, alternative decision-making strategies may be employed^7^,^8^. It has been suggested, for example, that when making decisions in which the outcome is uncertain, humans rely more on emotional than conscious thought processes^9^, to provide a rapid but crude appraisal of the available options^10^. This theory, known as the somatic marker hypothesis^9^, proposes that physiological responses which have previously been associated with the available options (stored as “somatic markers”) are recalled during this assessment period to aid decision-making. In humans, this process occurs before an individual is consciously aware of the “advantageous” or “disadvantageous” options^11^.

The neural mechanisms underlying decision-making under uncertainty have been investigated in humans to some extent (see ref. 12). A considerable effort has also been made to link these processes with measures of autonomic arousal (e.g. refs. 13,14,15), both in anticipation of and as a consequence of decision-making^16^. Although some work has begun to monitor neural processes during decision-making requiring risk assessment in non-human species (see review^17^), it is not known whether other animals also adopt rapid appraisal mechanisms, such as recalling arousal, when assessing risk. In a previous study, we found that behavioural and physiological measures of arousal were detectable when chickens made simple foraging decisions^18^, but it was not clear whether arousal influenced choice behaviour or whether it was simply a conditioned anticipatory response to food. In the current study we therefore aimed to monitor behaviour and physiology at different stages of decision-making, when individuals chose between a high-gain, high-risk option and a low-gain, no-risk option.

In the aforementioned human studies, risk perception was monitored by taking measures of sympathetic autonomic arousal (skin conductance reactivity and heart-rate (HR)) during a period in which subjects “pondered” the available options, before they had explicit knowledge of which was the profitable outcome^11^,^16^. Although the effect of risk perception on arousal has not yet been studied in chickens, increased arousal has been measured in response to a mild air-puff, which individuals also learned to avoid (Edgar et al. in prep). In the current study we therefore used a high-gain (HG) reward paired with the risk of an air-puff (RAP) as an aversive stimulus, to determine whether the perception of risk increased arousal during decision-making and how it influenced choice behaviour.

During a set of tests, individual hens were given a choice between the HGRAP option and one with a low-gain and no air-puff risk (LGNAP). We monitored indicators of sympathetic autonomic arousal (HR), heart-rate variability (HRV), peripheral body temperature, which are sensitive markers of reward anticipation^18^,^19^ and aversive situations^20^,^21^. These physiological measures, along with behavioural indicators of arousal (head movements, latency to choose)^18^,^22^,^23^, were taken during a viewing period when chickens were presented simultaneously with the HGRAP and LGNAP options. In addition, we took repeated measures of sympathetic autonomic arousal during a post-decision, pre-outcome (anticipation) period and again when chickens accessed their chosen outcome. This sequential monitoring enabled us to (i) identify precisely when arousal occurred in relation to decision-making and (ii) assess whether autonomic responses (and potentially their associated emotions) influenced choice outcome both immediately and in the subsequent trial.

## Methods

### Ethics Statement

All work was conducted under UK Home Office licence (30/2779) and had University ethical approval. We also conducted the study in compliance with ASAB ethical guidelines. The hens were rehomed to small responsible free-range holdings after the study.

### Experimental Set-up and Habituation

Twenty-eight Columbian Black Tail laying-hens were obtained at 17 weeks of age and were group-housed in a single room (see supplementary information for full details).

The experimental room, separated from the home room by a solid metal door and a corridor to prevent noise transfer, contained a T-maze apparatus. In brief, the T-maze consisted of two pens joined by a Perspex tunnel, which included four pulley-operated doors; a start-box, with removal wooden side panels and a pulley-operated entrance door was attached to the tunnel. In each pen, a feeder was fixed to the back wall; an air puff could be delivered when required through this feeder. See supplementary information for full details.

Habituation to HR monitoring and the T-Maze were carried out in parallel (see supplementary information). Throughout the habituation and training phase, hen progress was assessed using training criteria.

### Colour-cue training: Pre air-puff phase

Twenty-two hens satisfied all habituation criteria and were grouped according to how quickly they were ready for testing (into six groups of 2–4 hens). The day before testing each group of hens began, two pieces of A4 card were stuck to the back of the tunnel (red on left, blue on right) and hens were trained to associate the colour and side of the tunnel with receiving either 1 (low-gain: LG) or 4 (high-gain: HG) pieces of sweetcorn in the feeder. The colour associated with each quantity of sweetcorn was systematically varied so that within each testing group an equal number of hens received four pieces of sweetcorn on the right and left. When the colours had been introduced, each hen was given a set of nine trials (six unidirectional and three free) to strengthen the association. The latency to choose was recorded, as was the choice made during the free trials. If any hen chose the lower quantity side of the T-Maze more than once in the three free trials, additional training trials were conducted.

On the day after each group of hens had been individually trained to recognise the colour-cues, we assessed whether hen behaviour or physiology was affected by food quantity (before we introduced the air-puff) during a further set of 10 training trials. We food deprived the birds for 2 h and put on the ECG pads and HR monitor 15 min before training to allow the hen time to adapt to walking. The first two trials were unidirectional (one to either side of the T-Maze as a “reminder” of the colour cues), followed by eight free trials, to assess whether they remembered which colour was associated with HG. During each of the 10 trials, baseline physiological measures were taken for 10 s after the hen was first placed into the start-box (baseline period). The side panels of the start-box were then removed to reveal the coloured card on the inside of the Perspex tunnel and the individual was confined for a further 10 s (the viewing period). The tunnel door was then raised allowing the hen to enter the tunnel and make a choice by moving towards either of the pen doors. Once a choice had been made the relevant middle door was closed and the hen was confined between the middle and pen doors for 10 s (anticipation period) prior to the pen door being opened, allowing access to the feeder. Once the hen had reached the feeder, measures were taken during the first 10 s (reward period). The hen was kept within the pen after making a choice for 1 min, before the next test commenced. If a hen failed to leave the start-box after 60 s, she was gently encouraged to move into the tunnel. Similarly if she failed to enter the pen once the pen door had been removed for 120 s, she was gently encouraged into the pen.

Criteria were set (that hens chose HG at least 70% of the time) to progress to the testing phase. Although all hens showed a greater preference for the HG side, a few hens didn't quite reach criteria, so additional trials were given as necessary.

### Testing phase

Immediately after each individual completed the 10 pre air-puff trials, during the same session, each hen was given a set of 30 free-choice trials in which the HG side of the T-maze became the HGRAP option and the LG side became the LGNAP option. This was achieved by pairing HG with the risk of receiving a single air-puff at the feeder, at a fixed probability of 1 in 4. The air-puff schedule was predetermined for each hen, although criteria were set to prevent more than four air-puffs being delivered consecutively. The air-puff was delivered from outside the pen when the experimenter viewed the hen's head in the feeder. All other aspects of each trial were as described for the pre air-puff phase. If a hen made 10 consecutive choices to the same side, a unidirectional trial was given to the opposite side. During each trial, behaviour and physiology were recorded.

### Behavioural and physiological measures

The latency to reach the middle door (i.e. time from tunnel door being raised to the start of the anticipation period) and to the feeder (i.e. time from pen door being raised to reaching the feeder) were both recorded using a stop-watch. A CCTV camera was fixed above the start-box and video was continuously recorded using WebCCTV software. The number of head movements made during the viewing period was subsequently recorded using Windows Media Player. The decision outcome was also noted for each trial.

ECG was monitored as in^18^ using non-invasive remote telemetric units^24^ and cables contained within a harness. The monitor communicated with a base unit (attached to a computer via USB connection) and was controlled using RVC Telemetry Software version 1.5. Measures of HR and HRV were extracted using Spike 2 Software (version 6) from four 10 s periods: baseline, viewing, anticipation and reward. From each 10 s period, an average of HR (bpm) and two measures of HRV – the root mean square of the successive differences between beats (RMSSD) and the coefficient of variance (standard deviation of the mean interval between beats divided by the mean interval between beats – SDNN/RR) – were taken. Baseline measures were taken to control for individual differences at the start of the test. The percentage change in HR between the baseline and viewing periods, between the viewing and anticipation periods and between the anticipation and the reward periods were subsequently calculated and were analysed in addition to absolute values during each period.

Surface body temperature was recorded whilst hens were in the start-box using a thermal video camera (FLIR SC305). Eye and maximum head temperature data were extracted from a clear image during the baseline and viewing periods of each test using FLIR ResearchIR Software version 1.2 SP2. The percentage change in both eye and head temperature between the two periods was subsequently calculated and were analysed in addition to absolute values during the baseline and viewing periods.

### Statistical Analysis

Data were analysed using *IBM SPSS Statistics 21*. For each set of data, the assumptions of parametric testing were checked and data were transformed if possible, then analysed using paired-samples t-tests. Where transformations were not possible or unsatisfactory, Wilcoxon tests were used. Because multiple t-tests were conducted on behavioural and physiological data collected from the same hens during the same testing periods, a Benjamini-Hochberg correction was applied to relevant *p*-values from each testing period. There were occasional missing data when HR or temperature could not be obtained. Unless otherwise stated, means ± SE are presented throughout and a measure of effect size is given alongside significant results.

Twenty-one hens completed both the pre air-puff training and the testing phases of the experiment. The pre air-puff data were analysed to check for differences in behavioural and physiological measures of arousal, relating to food quantity (prior to air-puff introduction). An average was taken for each hen from trials when they chose HG and LG. Three hens chose only the HG option, so were excluded from these analyses. Bivariate correlations were also used to identify relationships between HG preference (proportion of times chosen) and individual hen's physiological responses during tests. The data were also analysed to monitor the effect of the choice in one trial on behavioural and physiological measures during the baseline and viewing periods in the subsequent test.

Data from the testing phase were analysed in the same way as the pre air-puff training data. Data were included only after hens had experienced their first air-puff. For each behavioural and physiological measure an average was taken for each hen from tests in which they chose HGRAP and from tests in which they chose LGNAP. We also checked for differences in baseline and viewing period measures in those trials that immediately *followed* an HGRAP choice that had resulted in an air-puff, to identify whether this influenced arousal. Most hens didn't experience more than three or four air-puffs, but a few hens experienced up to nine. Five hens continuously chose HGRAP throughout the testing phase, hence they were excluded from this part of the analyses. Additionally, bivariate correlations were used to identify relationships between HGRAP preference (proportion of times chosen) and individual hen's physiological responses during tests.

## Results

### Pre air-puff phase

During the pre air-puff phase, individual hens chose the HG option on average 82 ± 2% (range: 70–100%) of the time. There were no significant differences in any of the indicators of arousal (latency to choose or to feeder, head movements, HR, HRV and temperature) during trials when hens chose the HG compared with the LG option (paired samples t-tests: all *p* > 0.05). However, the proportion of times hens chose the HG option during the pre air-puff phase was significantly, positively correlated with the HR change between the viewing and anticipation periods (Pearson correlation coefficient = 0.45, *p* = 0.040, *n* = 21) and was negatively correlated with the change between the waiting and reward periods (Pearson correlation coefficient = −0.49, *p* = 0.025, *n* = 21). All other physiological variables showed no significant correlation with the proportion of times HG was chosen.

There were no significant differences in physiological and behavioural measures (HR, temperature, HR and temperature change, head movements) during the baseline or viewing periods in the trial following an HG or LG choice outcome in the previous trial (paired samples t-tests: all *p* > 0.05).

### Air-puff testing phase

During the testing phase, individual hens chose the HGRAP option on average 64 ± 7% (range: 12–100%) of the time.

#### Were there any significant differences in arousal during trials when hens chose the HGRAP compared with the LGNAP option?

**Figure 1:**
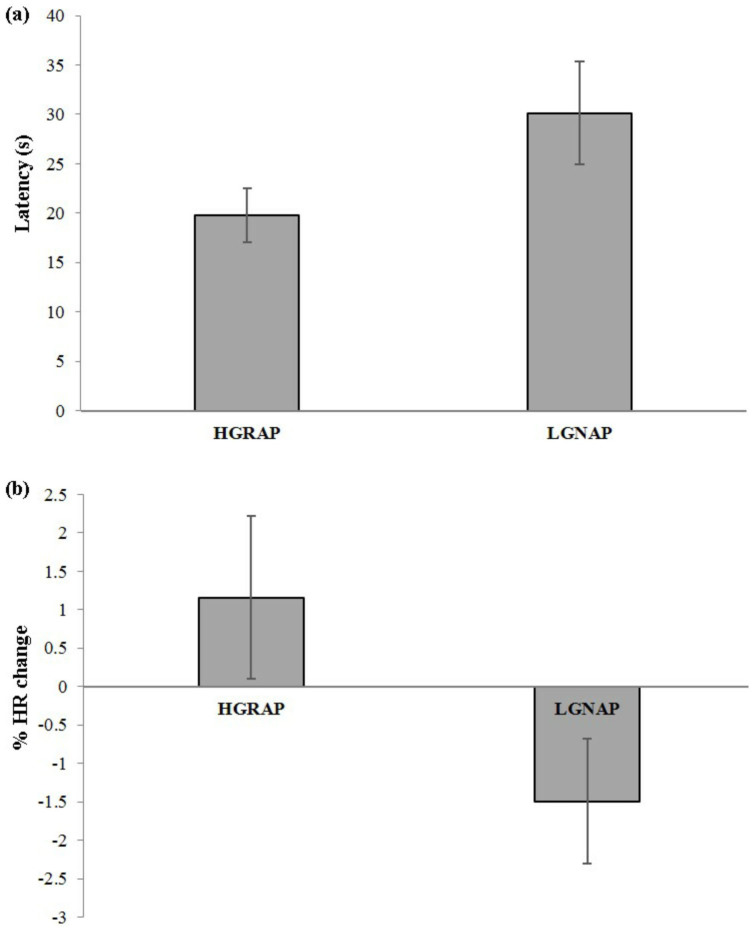
Mean ±1 SE (a) latency to anticipation period (*n* = 16, *p* = 0.035) and (b) percentage change in HR between the viewing and anticipation periods (*n* = 16, *p* = 0.012) when choosing the HGRAP and LGNAP options.

The majority of physiological and behavioural measures taken during the baseline, viewing anticipation and pen periods (HR, temperature, HR and temperature change, head movements) did not differ significantly between trials when hens chose HGRAP compared with LGNAP (supplementary table 1). However, significantly lower latencies to choose (paired samples t-test: *t*_15_ = 2.32, *p* = 0.035, eta squared = 0.26, figure 1a), a greater HR change between the viewing and anticipation period (*t*_15_ = 2.85, *p* = 0.012, eta squared = 0.35, figure 1b) and a significantly higher reward period HR (*t*_15_ = 2.80, *p* = 0.039, eta squared = 0.35, figure 1c) were apparent when hens chose the HGRAP option.

#### Were there significant correlations between the proportion of times HGRAP was chosen and the physiological responses during tests?

There were significant, positive correlations between the proportion of times HGRAP was chosen after the first air-puff had been received and the HR during the baseline (Pearson correlation coefficient = 0.61, p = 0.003, n = 21), viewing (Pearson correlation coefficient = 0.67, p = 0.001, n = 21), anticipation (Pearson correlation coefficient = 0.60, p = 0.004, n = 21) and reward (Pearson correlation coefficient = 0.57, p = 0.007, n = 21) periods. There was also a significant negative correlation between the proportion of times HGRAP was chosen and SDNN/RR during the viewing period (Pearson correlation coefficient = −0.49, p = 0.028, n = 20). All other physiological variables showed no significant correlation with the proportion of times HGRAP was chosen.

#### Were there any significant differences in arousal at the start of the subsequent trial following a HGRAP or LGNAP outcome?

The majority of dependent variables (HR, temperature, HR and temperature change, latency, head movements) were not significantly different during the baseline and viewing periods of the next trial after hens had chosen LGNAP or HGRAP in the previous trial (supplementary table 2).

#### Were there any significant differences in arousal at the start of the subsequent trial following an air-puff outcome?

There were no significant differences in absolute physiological and behavioural measures (HR, temperature, head movements), or changes between baseline and viewing periods (HR and temperature), in the trials after hens received an air-puff compared with trials after a HGRAP choice that resulted in no air-puff being received (supplementary table 3).

#### Did the air-puff influence subsequent choice behaviour and arousal?

**Figure 2:**
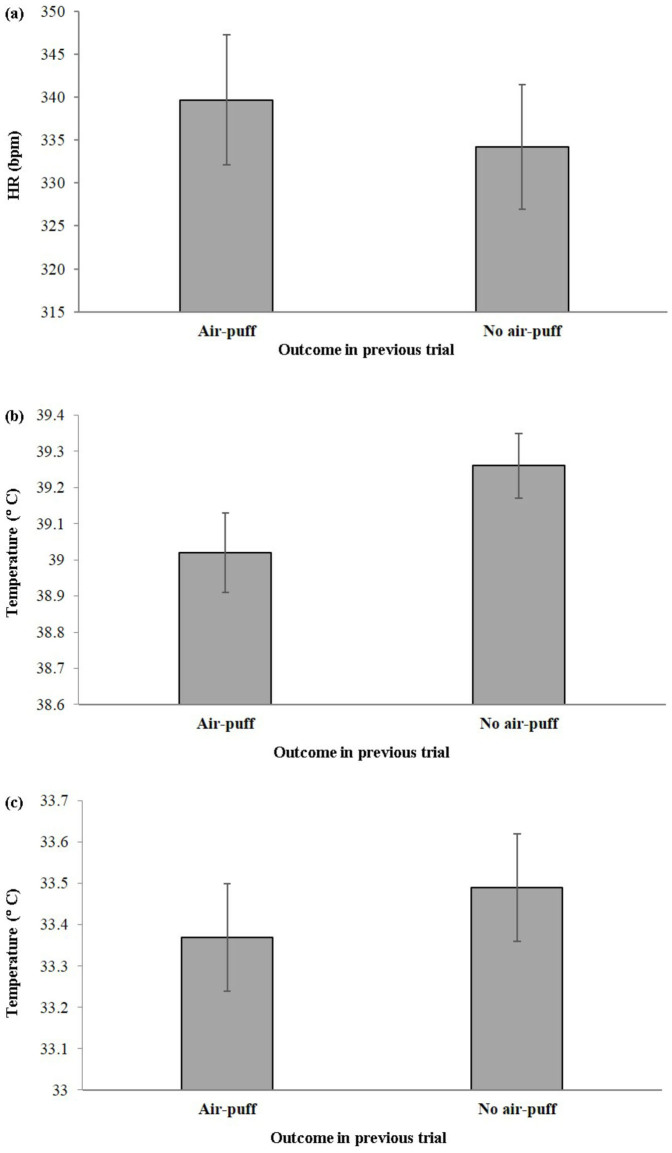
Mean ±1 SE (a) baseline HR (*n* = 16, *p* = 0.045), (b) maximum head temperature during the viewing period (*n* = 15, *p* = 0.035), and (c) eye temperature during the viewing period (*n* = 15, *p* = 0.033) when choosing the HGRAP option following HGRAP outcomes that did and did not result in an air-puff.

After an air-puff was received, there was no significant difference in the likelihood of HGRAP being chosen in the subsequent trial compared to when no air-puff was received (HGRAP (after air-puff): 57 ± 9%; HGRAP (after no air-puff): 65 ± 7%; paired samples t-test: *t*_20_ = 1.49, *p* = 0.153). No measures were significantly different when comparing choices of the HGRAP and LGNAP option in the trial following an air-puff (all *p* > 0.05). However, when hens had received an air-puff and chose HGRAP in their subsequent trial, their baseline HR in that subsequent trial was significantly higher than when no air-puff was received in the previous trial and HGRAP was chosen in the subsequent trial (*t*_15_ = 2.99, *p* = 0.045, eta squared = 0.37, figure 2a). Both the maximum head (*t*_14_ = 3.15, *p* = 0.035, eta squared = 0.39, figure 2b) and eye temperature (*t*_14_ = 2.84, *p* = 0.033, eta squared = 0.37, figure 2c) were significantly lower during the subsequent viewing period when HGRAP was chosen following an air-puff compared with no air-puff.

## Discussion

The role of physiological arousal or stress in decision-making is often invoked but has been little investigated in animals. By partitioning the decision-making process, and monitoring physiology and behaviour at each stage, we found that hens reacted to risk with elevations in physiological arousal, but this did not deter them from choosing high-gain outcomes associated with risk.

It was the perception of risk rather than reward that produced an elevation in HR during the anticipation period preceding each HGRAP choice. During the pre-air puff phase HR was not affected by reward magnitude, but during the testing phase arousal increased between the viewing and anticipation periods when hens chose HGRAP rather than LGNAP. There was no further significant increase between anticipation and reward periods. The only methodological difference between the testing phase and the pre air-puff phase was the risk of an air-puff. Although it has previously been shown that arousal increases in anticipation of conditioned appetitive and aversive events delivered with 100% contingency in chickens (e.g. refs. 18,19,20,21,22), our work shows that hens are sufficiently sensitive to anticipate a 25% risk of an aversive event. During the anticipation period, hens were therefore responding physiologically to their perception of the risk associated with the HG option.

In addition, several of our measures of arousal were influenced by the nature of the previous decision. During the testing phase, HR was significantly higher during the baseline period, and the maximum head and eye temperature during the viewing period were also significantly lower when HGRAP was chosen in a subsequent trial after an air-puff compared to when it was chosen after no air-puff. It is likely, therefore, that in the subsequent trial we were measuring a residual effect of the increased arousal caused by hens “coping” with the previous HGRAP outcome (e.g. ref. 25). These combined results suggest that receiving an air-puff has a measurable impact on physiological arousal in the subsequent trial.

One of our main findings was that none of the measures of arousal that were associated with the HGRAP outcome affected the birds' subsequent tendency to choose this same outcome. In short, arousal was not a good marker of avoidance. Although, in general, chickens seemed to reduce their preference for the HG option when it was paired with the risk of an air-puff, they showed no decreased probability of choosing it directly after receiving an air-puff. This was despite increased arousal being evident at the time when a choice was being made. Additionally, the proportion of times HGRAP was chosen during the testing phase was significantly, positively correlated with HR during all phases of the test, suggesting that more persistent birds did find the air-puff arousing. It would seem, therefore, that the function of arousal was not in mediating decision-making, but was likely associated with both reward activation and punishment-avoidance systems. Arousal may prepare an individual for fight or flight (e.g. ref. 26) and is not necessarily negative. In this case, the birds that continued to choose the HGRAP option clearly experienced it as positively valenced (e.g. ref. 27). We do not know how individuals made the assessment to reduce their preference for the HGRAP option, but possibly high arousal is recalled during later assessments and is used in conjunction with other information to produce the overall decline in visits.

This analysis leads to an interesting comparison with the somatic marker hypothesis, which proposes that arousal is generated and used (subconsciously) in humans to indicate options that should be avoided^9^,^11^. It seems from our work that arousal generated during decision-making is not always used as a marker signifying that an option should be avoided. One possibility is that arousal must be accompanied by some other assessment and coding of long-term gain or loss for it to influence decision-making. A key difference between the Iowa Gambling Task (IGT), which is used to test human decision-making, and the task we developed here is that the IGT produces immediate gains and losses, which lead to long-term advantageous and disadvantageous options^28^. Anticipatory responses are generated prior to choosing the disadvantageous option which humans ultimately learn to avoid (e.g. ref. 11). The options that we presented had no clear advantageous or disadvantageous long-term consequences and although some individuals showed a preference for HGRAP or LGNAP, these individual preferences likely depended on how well each individual coped with the aversive air-puff stimulus. Possibly arousal must be mediated by some neural coding of loss before it is used as a marker that an outcome should be avoided.

Just as arousal does not always lead to avoidance, not all types of ‘difficult' decision lead to arousal. For example, arousal is no greater when hens have to choose between finely balanced (options of equal net value) than when they have to choose between options of unequal net value^29^. We suggest that a broad range of decision-tasks should be used before general conclusions can be drawn about the role of physiology in decision-making.

As an additional point, we noted that hens were significantly quicker to make their choice when choosing HGRAP over LGNAP. Heightened arousal (HR) when choosing HGRAP could have resulted in hens approaching the door more quickly or they may have taken less time to consider a HGRAP choice outcome than LGNAP. Alternatively, hens might have been very committed to choosing the HGRAP option and therefore decided more quickly. It has previously been found that chickens with strong and consistent preferences had shorter latencies to choose^30^.

In summary, we have found increased arousal in anticipation of the risk associated with the high-gain option. We also found that receiving an air-puff resulted in increased arousal during the subsequent trial, but only when the HGRAP option was chosen again. Interestingly, however, high arousal did not result in hens avoiding the HGRAP option, suggesting that it is not a good measure of avoidance. Although we found that some measures of arousal increased after hens had received an air-puff, our results suggest that this was likely to be a residual effect of the air-puff rather than arousal (somatic markers) mediating subsequent decision-making as seen in human decision-making tasks. We suggest that for elicitation of somatic markers, choice outcomes must be differentiable in long-term gain or loss.

## Author Contributions

A.D., A.R. and C.N. wrote the main manuscript text. A.D., A.R. and C.N. designed the experiment. A.D. and I.P. performed the experiments. A.D. and F.Y. extracted the data and A.D. analysed the data. All authors reviewed the manuscript.

## Supplementary Material

Supplementary Information Supplementary information
