# Supplementary material for: Elevated arousal at time of decision-making is not the arbiter of risk avoidance in chickens
Source: Sci Rep. 2015 Feb 3;5:8200. doi: 10.1038/srep08200 (PMC4314631; doi:10.1038/srep08200)
Supplement: Supplementary Information [file srep08200-s1.pdf]

ELEVATED AROUSAL AT TIME OF DECISION-MAKING IS NOT THE ARBITER OF RISK AVOIDANCE IN CHICKENS

A.C. DAVIES, A.N. RADFORD, I.C. PETTERSSON, F.P. YANG & C.J. NICOL

Supplementary Information

Supplementary Figure 1. T-maze test apparatus consisting of a Perspex tunnel and an attached wooden start-box. The tunnel connects the two pens in the experimental room. **A** indicates the wooden side panels which were removed to reveal wire mesh, through which the coloured card on the inside of the Perspex could be viewed. **B** indicates the tunnel-door, which was raised using a pulley mechanism to allow access to the tunnel. **C** marks the middle doors which were closed once the hen had passed beneath it to confine the hen for the anticipation period. **D** marks the pen-door which was in the closed position until the end of the anticipation period, after which the hen was allowed to enter the pen. Once the hen entered the pen the door was replaced to prevent her from re-entering the tunnel. **E** marks the feeder at the back of the pen where the air-puff was also administered.

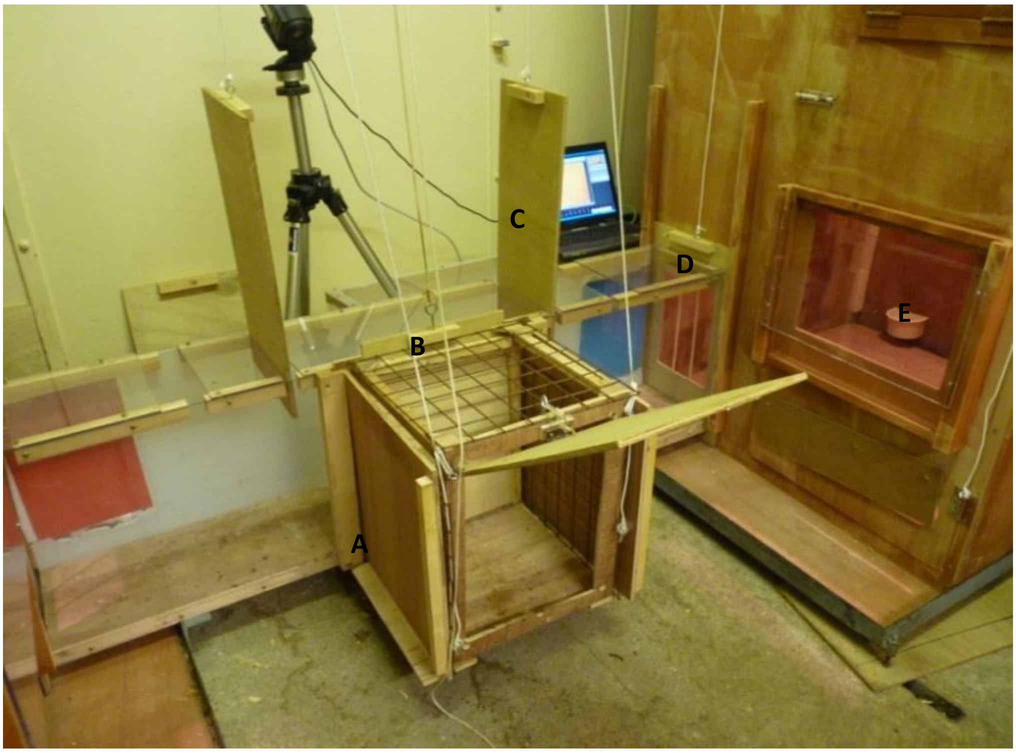

RESULTS

Supplementary Table 1. Physiological and behavioural measures tested for significant differences between HGRAP and LGNAP choice outcomes during different periods within trials.

Measure	HGRAP Mean ± SE	LGNAP Mean ± SE	*df*	*P* value	Effect size
**Baseline Period**					
HR	318 ± 7 bpm	322 ± 7 bpm	15	0.830	-
RMSSD	5.2 ± 0.6 ms	6.2 ± 1.4 ms	14	0.571	-
SDNN/RR	0.037 ± 0.002	0.039 ± 0.004	14	0.681	-
Eye temperature	33.3 ± 0.2 °C	33.4 ± 0.2 °C	15	0.498	-
Max head temperature	39.2 ± 0.1 °C	39.1 ± 0.1 °C	15	0.353	-
**Viewing Period**					
HR	310 ± 7 bpm	314 ± 7 bpm	15	0.530	-
RMSSD	7.1 ± 0.7 ms	6.2 ± 0.8 ms	14	0.635	-
SDNN/RR	0.044 ± 0.003	0.039 ± 0.003	14	0.445	-
Head movements	6.8 ± 0.4	6.3 ± 0.3	15	0.212	-
Eye temperature	33.4 ± 0.2 °C	33.4 ± 0.2 °C	15	0.793	-
Max head temperature	39.1 ± 0.1 °C	39.2 ± 0.1 °C	15	0.476	-
**Anticipation Period**					
HR	313 ± 8 bpm	309 ± 7 bpm	15	0.465	-

RMSSD	5.6 ± 0.8 ms	5.1 ± 0.9 ms	14	0.645	-
SDNN/RR	0.034 ± 0.003	0.033 ± 0.003	14	0.618	-
**Reward Period**					
HR	322 ± 7 bpm	313 ± 6 bpm	15	**0.039**	0.35
RMSSD	6.0 ± 0.9 ms	6.1 ± 0.9 ms	14	0.921	-
SDNN/RR	0.044 ± 0.004	0.043 ± 0.003	14	0.788	-
**Change between baseline and viewing period**					
HR	-2.7 ± 0.4%	-2.5 ± 0.3%	15	0.942	-
Eye temperature	0.4 ± 0.4%	0.3 ± 0.3%	15	0.970	-
Max head temperature	0.0 ± 0.2%	0.3 ± 0.2%	15	0.645	-
**Change between viewing and anticipation period**					
Latency to choose	19.8 ± 2.7 s	30.1 ± 5.2 s	15	**0.035**	0.26
HR	1.2 ± 1.1%	-1.5 ± 0.8%	15	**0.012**	0.35
**Change between anticipation and reward period**					
Latency to feeder	10.0 ± 2.6 s	13.1 ± 6.6 s	15	0.929	-
HR	3.1 ± 1.0%	1.7 ± 0.8%	15	0.191	-

Supplementary Table 2. Physiological and behavioural measures tested for significant differences following HGRAP and LGNAP choice outcomes during the subsequent baseline and viewing period.

**Measure**	**HGRAP Mean ± SE**	**LGNAP Mean ± SE**	***Df***	***P* value**	**Effect size**
**Baseline Period**					
HR	323 ± 6 bpm	320 ± 7 bpm	15	0.617	-
RMSSD	6.0 ± 0.8 ms	5.50 ± 1.1 ms	14	0.710	-
SDNN/RR	0.042 ± 0.003	0.036 ± 0.003	14	0.105	-
Eye temperature	33.2 ± 0.2 °C	33.3 ± 0.2 °C	15	0.918	-
Max head temperature	39.1 ± 0.1 °C	39.1 ± 0.1 °C	15	0.854	-
**Viewing Period**					
HR	314 ± 6 bpm	310 ± 6 bpm	15	0.670	-
RMSSD	6.1 ± 0.6 ms	6.2 ± 0.8 ms	14	0.858	-
SDNN/RR	0.041 ± 0.002	0.040 ± 0.002	14	0.711	-
Head movements	6.5 ± 0.4	6.3 ± 0.4	15	0.423	-
Eye temperature	33.3 ± 0.2 °C	33.4 ± 0.2 °C	15	0.503	-
Max head temperature	39.2 ± 0.1 °C	39.1 ± 0.1 °C	15	0.733	-
**Change between baseline and viewing period**					
HR	-2.8 ± 0.5%	-2.8 ± 0.5%	15	0.985	-
Eye temperature	0.3 ± 0.2%	0.3 ± 0.3%	15	0.818	-

Max head temperature	0.2 ± 0.1%	0.0 ± 0.2%	15	0.906	-

Supplementary Table 3. Physiological and behavioural measures tested for significant differences following HGRAP choices that did and did not result in an air-puff, during the subsequent baseline and viewing period.

**Measure**	**HGRAP Air-puff Mean ± SE**	**HGRAP No air-puff Mean ± SE**	***df***	***P* value**	**Effect size**
**Baseline Period**					
HR	326 ± 6 bpm	320 ± 7 bpm	15	0.478	-
RMSSD	5.9 ± 0.9 ms	6.0 ± 1.1 ms	14	0.901	-
SDNN/RR	0.043 ± 0.004	0.040 ± 0.004	14	0.712	-
Eye temperature	33.1 ± 0.2 °C	33.3 ± 0.2 °C	15	0.445	-
Max head temperature	39.1 ± 0.1 °C	39.1 ± 0.1 °C	15	0.950	-
**Viewing Period**					
HR	316 ± 6 bpm	311 ± 7 bpm	15	0.683	-
RMSSD	5.8 ± 0.9 ms	6.2 ± 0.6 ms	14	0.622	-
SDNN/RR	0.039 ± 0.004	0.041 ± 0.002	14	0.698	-
Head movements	6.4 ± 0.5	6.5 ± 0.4	15	0.831	-
Eye temperature	33.2 ± 0.2 °C	33.3 ± 0.2 °C	14	0.753	-
Max head temperature	39.1 ± 0.1 °C	39.2 ± 0.1 °C	14	0.535	-

Change between baseline and viewing period					
HR	-3.0 ± 0.6%	-2.9 ± 0.5%	15	0.890	-
Eye temperature	0.3 ± 0.4%	0.1 ± 0.3%	14	0.779	-
Max head temperature	-0.1 ± 0.2%	0.3 ± 0.1%	14	0.564	-

METHODS

Animals, Housing and Husbandry

Hens were group-housed in a room (3.05 x 3.66 m) containing a two-tiered bank of 10 nest boxes (individual nest box dimensions: 0.26 x 0.35 x 0.36 m), three, two-tiered perches (each length of perch was 0.85 m) and were bedded with approximately 10 cm of wood-shavings. *Ad libitum* feed (Farmgate Layers Mash, BOCM Pauls, Ipswich, Suffolk, UK) was provided in each room via a large suspended feeder (0.4 m diameter) with 16 individual compartments and water was provided via a hanging drinker (0.38 m diameter). The room temperature was kept at 18–22°C and the lighting schedule was 12 L : 12 D (light period 7 am – 7 pm).

Experimental Room

The experimental room had two pens (1 x 1 m), one on each side of the room. The pens were joined by a Perspex tunnel (1.54 x 0.24 m, 0.47 m high), which formed a T-Maze apparatus when a start-box (0.38 x 0.39 m, 0.47 m high) was attached. The pens were made of solid wood, with the exception of a Perspex window (33 x 26 cm) on the front of both pens, to allow the experimenter to see into the pen. The start-box had removable wooden side panels and a pulley-operated tunnel entrance door. Inside the tunnel, four further pulley-operated

doors (two either side) could be inserted as necessary (see supplementary figure 1). All doors were solid wood with the exception of the pen doors which had an open gap (0.18 x 0.27) containing two vertical wooden rods (with 5.5 cm gaps between them). A feeder was attached to the back wall of each pen (0.28 m from the pen side). Each feeder had a small hole (3 cm from the base) from which a tube (attached to the inside of the feeder) ran to the outside of the pen (internal diameter: 35 mm, length: 4.5 m). This allowed for an air canister (AF PC Spray Duster Invertible) to be attached to the external end of the tubing, so that an air-puff could be administered from outside the pen as necessary.

An additional room (holding room) with the same dimensions as the home and experimental rooms was used for food deprivation and HR monitor habituation. This room contained shavings (5–10 cm deep) and two bell-drinkers (25 cm diameter).

Habituation to Handling, HR monitoring and T-Maze

Hens were firstly habituated to human presence and handling (days 1–3), then to moving through the T-Maze apparatus and feeding from the experimental feeders in groups (days 3–6) and finally individually (days 6–28). Habituation to the harness containing the HR monitor was conducted firstly in groups (in a separate room) (days 6–25), then individually during T-Maze habituation (days 25–28). During individual T-Maze habituation, only one arm of the T-maze apparatus was open on any given day, but experience with both arms was balanced across days. Habituation and training took 6–7 weeks, depending on individual progression. In total during the habituation period, between 52 and 65 unidirectional trials were carried out until the majority of hens wore the harness whilst moving through the apparatus without hesitation or distress and ate from the feeders on both sides of the T-Maze.
